# Long-Term Outcomes of the Ovation Stent Graft System: Single-Center Experience

**DOI:** 10.3390/jcm14124177

**Published:** 2025-06-12

**Authors:** Gianluigi Fino, Giacomo Isernia, Gianbattista Parlani, Adriana Belardi, Francescopio Del Mastro, Enrico Cieri, Massimo Lenti, Gioele Simonte

**Affiliations:** Vascular and Endovascular Surgery Unit, S. Maria della Misericordia University Hospital, 06132 Perugia, Italy; iserniagiacomo1@gmail.com (G.I.); parlani.gianbattista@gmail.com (G.P.); adrianabelardi@gmail.com (A.B.); francescopidelmastro@gmail.com (F.D.M.); enrico.cieri@unipg.it (E.C.); massimo.lenti@gmail.com (M.L.); giosimonte@gmail.com (G.S.)

**Keywords:** endograft, endovascular aortic repair, EVAR, low profile, hostile access, hostile neck

## Abstract

**Background/Objective**: To report mid-term to long-term outcome data for endovascular aortic repair using the Ovation stent graft system (Endologix, Santa Rosa, CA) for the correction of abdominal aortic aneurysms (AAAs) in a single center. **Methods**: All patients treated with the Ovation stent graft between December 2011 and February 2018 were included. Patient demographics, anatomical and operative details, as well as follow- up data including complications, the need for further interventions, and mortality were recorded prospectively in an electronic dataset and analyzed. **Results**: A total of 99 patients (86.10% males; mean age 73.6 ± 7.26 years) were treated with the Ovation stent graft. The mean maximal aortic diameter was 53.7 ± 8.8 mm mm. The main indications for Ovation use were small iliac accesses and thrombus/calcification at the proximal neck level. The technical success rate was 93.06%. No perioperative reintervention or limb occlusion was reported. Two graft-related perioperative adverse events were recorded. At a mean follow up of 82.70 ± 40 months, cumulative late survival was 97.90%, 92.60%, 81.00%, 73.40%, 48.70%, and 45.10%, respectively, at 12, 24, 48, 60, 108, and 120 months. No AAA-related death was recorded. Actuarial freedom from reintervention rate was 97.90%, 95.70%, 92.10%, and 80.10%, respectively, at 12, 24, 60, 108, and 120 months; estimated freedom from conversion was 98.90%, 97.70%, and 95.20% at 24, 60, 108, and 120 months. **Conclusions**: The Ovation stent graft demonstrated durable AAA exclusion even in complex anatomies evidenced by successful aneurysm exclusion and mid- to long-term freedom from aneurysm-related mortality. However, in this series, the not insignificant graft-related adverse event rate suggested the need for structural improvements, which were implemented in the next-generation devices.

## 1. Introduction

Endovascular aneurysm repair (EVAR) is considered the mainstay of treatment of abdominal aortic aneurysms (AAAs), the prevalence of which ranges from 1% to 12.7% [1,2]. Randomized trials, meta-analyses, and large observational studies have repeatedly demonstrated that EVAR has lower perioperative mortality and morbidity than open repair [3]. However, approximately 50% of patients with AAAs requiring treatment are not eligible for on-label EVAR because of challenging anatomy [4,5,6]. The most common issues are short neck length, small access vessel diameter, and excessive neck angulation [7,8]. Safely expanding treatment eligibility and improving repair durability are the most important challenges to be solved in elective EVAR. Ovation, Ovation Prime, Ovation iX, and finally Ovation Alto have already proven to have safe outcomes with very satisfactory early and mid-term results [9,10,11,12]. Until now, long-term outcome results and comparative studies have been poor [13]. The aim of this retrospective study is to present our single-center mid-term to long-term outcome results with the use of the Ovation, Ovation Prime, and Ovation iX stent grafts.

## 2. Materials and Methods

All patients treated with the Endologix Ovation platform (including Ovation, Ovation Prime, and Ovation iX Abdominal stent graft systems) from December 2011 to February 2018 in a single-center university hospital were retrospectively analyzed from a prospective database. All patients gave informed consent before the EVAR procedure. Written consent was obtained from patients for anonymous usage of data and images for scientific purposes.

Intervention was indicated for patients presenting with infrarenal abdominal aortic aneurysms measuring ≥50 mm in diameter, common iliac artery aneurysms ≥35 mm, or infrarenal penetrating aortic ulcers.

Aneurysm morphology and visceral vessel characteristics were evaluated using thin-slice computed tomography angiography (CTA) with specialized reconstructions on dedicated workstations (Aquarius Terarecon, Foster City, CA, USA). Patients were considered to have a hostile aortic neck anatomy if their infrarenal neck angulation was ≥60, neck length <10 mm, infrarenal aortic diameter < 16 mm or >30 mm, and an unfavorable neck shape, such as conical, reversed conical, >50% calcification, and circular or semicircular thrombus.

All interventions were conducted by an experienced vascular surgery team under local or general anesthesia, adhering to standardized operative protocols. Procedures were carried out in a hybrid operating suite outfitted with a fixed X-ray system and flat-panel detector. A final angiogram was systematically performed to verify graft patency and complete aneurysm exclusion.

Intraoperative outcomes were assessed based on technical success, the need for additional procedures, the requirement for surgical conversion of access sites, the presence of endoleaks on the final angiogram, limb ischemia, bleeding complications, and endograft occlusion.

According to the most recent reporting standards [14], technical success was defined as successful advancement and deployment of the endograft, without intraoperative mortality, conversion to open surgery, type I or III endoleak on completion angiography, or graft and limb occlusion.

Follow up consisted of a duplex scan at discharge and a contrast-enhanced CT within 30 days. Patients then underwent duplex surveillance at 6 months, a follow-up CT at 1 year, and annual duplex imaging thereafter. Additional CT was performed when clinically indicated (e.g., persistent endoleak, short proximal neck, sac growth > 5 mm). Follow-up data were obtained from scheduled outpatient evaluations conducted either at the treating hospital or at any external medical institutions. Patients without documented follow up for more than 12 months were contacted, and their records were updated based on telephone interviews. Causes of death were verified by the Regional Death Registry or by contacting the treating physician and patients’ families.

### 2.1. Statistical Analysis

Statistical analyses were conducted using SPSS Statistics software (version 30, IBM Corp, Chicago, IL, USA). Continuous variables were summarized as mean ± standard deviation (SD), while categorical data were reported as absolute numbers and percentages. The Kaplan–Meier estimate was used to define survival and reintervention during follow up. Estimates were considered reliable when the standard error was <0.10. A *p* value ≤ 0.05 was considered statistically significant in all analyses.

### 2.2. Device Features

The Ovation stent graft (Endologix, Irvine, CA, USA) has been commercially available in the United States since 2012. A unique feature of this device is its ability to accommodate a wider range of aortoiliac features, to navigate through complex iliac and femoral access, and to provide a seal in complex proximal infrarenal aortic neck morphology. The Ovation stent graft consists of a trimodular design with the aortic body delivered through a flexible hydrophilic coated 14 F outer diameter catheter. The aortic body is composed of a low-permeability polytetrafluoroethylene (PTFE) graft. Fixation is accomplished supra-renally using long rigid nitinol anchoring stents, which secure the device at the level of the superior mesenteric artery (SMA). Sealing is achieved through a series of inflatable channels and rings incorporated into the main body, free of metal components. During deployment, these channels are filled with a low-viscosity, radiopaque polymer that polymerizes in situ, conforming precisely to the patient-specific aortic neck anatomy, regardless of its shape and length. The Ovation iliac limbs are composed of highly flexible spiral nitinol stents encapsulated in low-permeability PTFE that are packaged in a low-profile 13 F or 14 F outer diameter delivery system. A detailed description of the device is included in the Ovation stent graft IFU [15].

## 3. Results

Throughout the study period, 99 patients (mean age, 73.6 years ± 7.26) were treated with the Endologix Ovation platform for infrarenal aortic disease. A total of 86.1% were male (87/99). The mean axial diameter of the treated aneurysm was 53.7 ± 8.8 mm. No ruptured cases were treated with this stent graft system. Demographic data, cardiovascular risk factors, and preoperative comorbidities are reported in Table 1.

Some features, considered as indicators of complex anatomy, were extensively prevalent in the population study. The main indications for Ovation use were small iliac accesses and thrombus/calcification at the proximal neck level. Moderate to severe iliac calcifications were detected in 44.55% of cases (45/99); complex proximal neck (length less than 10 mm, consistent thrombus or calcification, conical or reverse conical shape) was recorded in 45.54% of the cases (46/99). Baseline aneurysm characteristics are reported in Table 2.

**Figure 1:**
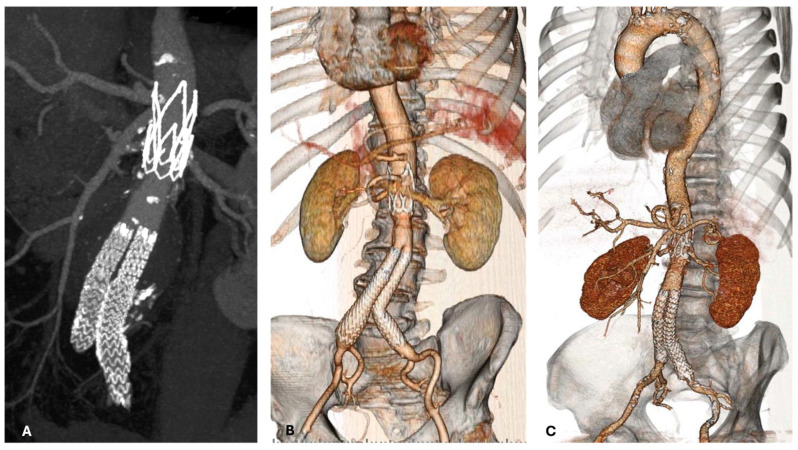
Postoperative CT angiogram demonstrating effective Ovation implantation and aneurysm exclusion in short neck (**A**,**B**) and in case of calcified iliac accesses (**C**).

Technical success was achieved in 93.06% of procedures (Figure 1). Three out of ninety-nine patients needed preoperative coiling of a hypogastric artery to create a safe distal sealing zone in the external iliac artery. Despite additional measures such as ballooning, three patients had a persistent type IA endoleak on completion of angiography. In this group, one patient received an aortic cuff Endurant (Medtronic, Santa Rosa, CA, USA), and two patients received a covered balloon expandable CP stent (NuMED, Inc., Hopkinton, NY, USA), with the resolution of the proximal leak in two out of three cases assessed by contrast-enhanced CT angiography (CTA) at 30 days.

One patient underwent embolization of the left renal artery in preparation for a scheduled nephrectomy. Another patient required correction with a bare-metal stent for segmental dissection of the right external iliac artery and ipsilateral femoral expansion patch due to hostile iliac–femoral access. Two perioperative complications related to the graft were recorded. In one case, a defect in the ring-filling mechanism led to abnormal bulging, resulting in significant stenosis at the proximal neck. In another case the polymer leaked into the blood flow with anaphylactic reaction and lumbar embolization. Procedural data are reported in Table 3.

Perioperative death occurred in three patients (one visceral ischemia, one myocardial infarction, and one ascending aortic aneurysm rupture). No perioperative AAA-related reintervention nor limb occlusion was reported.

During the follow-up period, five patients underwent embolization of type II endoleak at 9, 33, 75, 99, and 117 months, respectively, after the index procedure.

Four patients underwent iliac relining due to distal sealing failure during the follow-up period. In one patient, the correction was performed through the implantation of an iliac side branch at 77 months after the index procedure. In the other three patients, the correction was carried out by implanting an iliac cuff, with external iliac artery landing achieved in only one case, followed by embolization of the ipsilateral hypogastric artery (at 12, 24, and 81 months, respectively, after the index procedure).

One patient underwent embolectomy of the right iliac–femoral axis due to branch occlusion at 76 months after the index procedure. For the same reason, another patient underwent iliac stenting and femoro-femoral cross-over bypass at 32 months after the index procedure, followed by a left extra-anatomic axillo-monofemoral bypass due to occlusion of the femoro-femoral cross-over bypass at 62 months after the previous procedure.

In one case, two years after aortic endografting, a patient was treated in an emergency setting with an off-the-shelf branched endograft for acute aortic syndrome due to an 85 mm thoracoabdominal pseudoaneurysm involving the visceral arteries’ origin. An aortic wall injury that was possibly suprarenal stent-mediated appeared the most reliable etiology in this case. This patient also underwent thrombectomy and bilateral renal stenting approximately 8 months after the thoraco-abdominal procedure, followed by right iliac relining using the double barrel technique at 113 months after the index procedure.

In another case, a patient underwent a complex endovascular procedure using an off-the-shelf branched endograft for a penetrating juxtavisceral aortic ulcer at 130 months after the index procedure.

**Figure 2:**
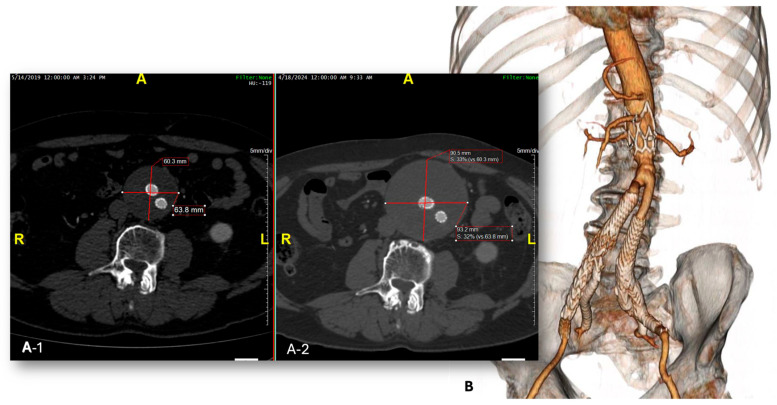
Control CTA showing evident enlargement of the aneurysmal sac due to type II endoleak (**A1,A2**). Partial surgical conversion with explantation of the endograft main body and preservation of the iliac side branches at 103 months after the index procedure (**B**).

During the follow-up period, three complete and one partial conversion were performed at 4, 36, 103, and 114 months after the index procedure, respectively (Figure 2).

In all cases, a growth of the residual aneurysmal sac due to a type II endoleak was detected. The early conversion at 4 months was performed in an emergency setting due to a contained rupture of the residual aneurysmal sac. Indications for reintervention are reported in Table 4.

The mean follow-up observational period was 82.73 ± 40 months (range 1–154 months). A total of 54 patients died during follow up. No AAA-related death was recorded.

**Figure 3:**
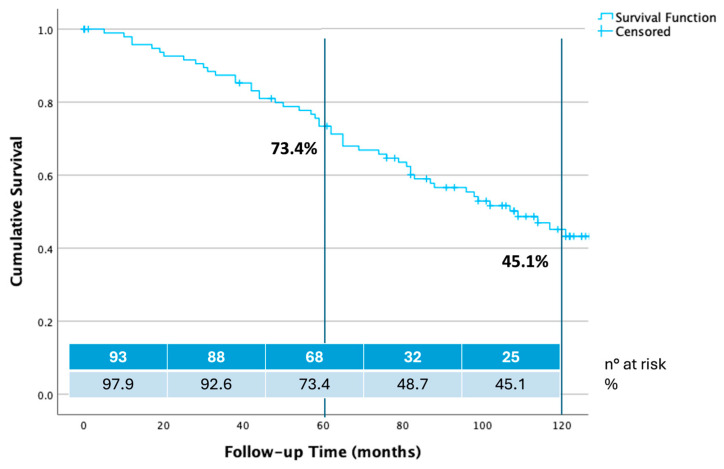
Ten years overall survival estimate calculated by Kaplan–Meier method.

Estimated freedom from all causes of mortality was 97.90% at 12 months, 92.60% at 24 months, 81.00% at 48 months, 73.40% at 60 months, 48.70% at 108 months, and 45.10% at 120 months (Figure 3).

**Figure 4:**
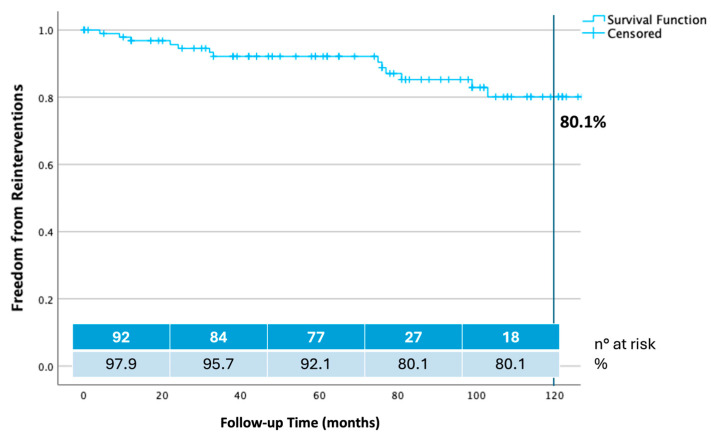
Ten years freedom from any reintervention calculated by Kaplan–Meier method.

Estimate of freedom from any reintervention was 97.90% at 12 months, 95.7% at 24 months, 92.10% at 60 months, and 80.10% at 108 and 120 months (Figure 4).

**Figure 5:**
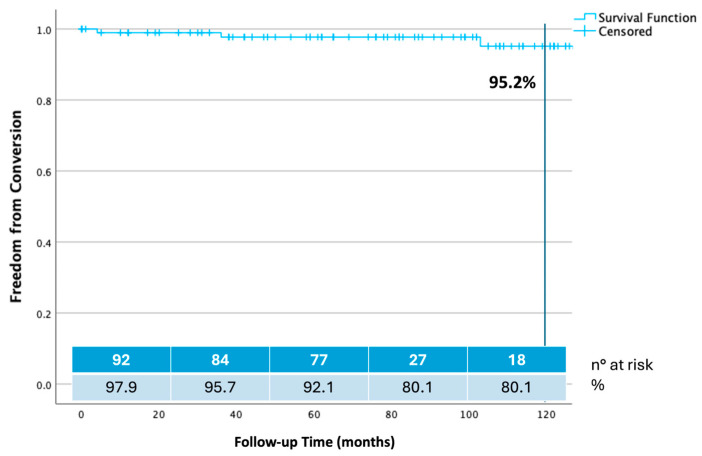
Ten years freedom from open surgical conversion calculated by Kaplan–Meier method.

Estimate freedom from conversion was 98.90% at 24 months, 97.70% at 60 months, and 95.20% at 108 and 120 months (Figure 5).

## 4. Discussion

In an era of increasing interest, expertise, and positive outcomes in complex fenestrated/branched EVAR for short-necked aortic aneurysms, the Ovation technology redefines the concept of a hostile neck for standard EVAR [16,17]. As EVAR technologies continue to advance, there remains significant potential for innovation to further enhance access to EVAR and ensure long-term durable aneurysmal exclusion.

The Ovation device is an endograft that warrants the physician’s attention due to its distinctive sealing mechanism and ability to accommodate challenging aortic neck and iliac anatomies. The use of the Ovation stent graft should be considered in cases of thrombotic apposition in the aortic neck or insufficient aortic neck length, particularly when the patient is not a candidate for open repair and complex endovascular repair is not a viable option. It may also be considered particularly suitable for cases involving small iliac arteries. Compared with other commercially available stent grafts, the Ovation device offers two key features that expand its treatment indications. First, the low-profile 14 F outer diameter (12 F inner diameter) delivery system enables access through narrow iliac arteries, giving it the smallest profile among all currently available stent grafts. The benefits of this design are undisputable, as 44.55% of patients enrolled in this cohort had a hostile iliac access. It is evident that the small profile of the Ovation device will broaden its applicability in pEVAR, replacing the conventional femoral cut-down and eliminating the associated complications [18]. While the Ovation device has demonstrated its potential through a continuous expansion of patient subpopulations, it simultaneously highlights the need for more comprehensive data in the medium and long term [13]. In this cohort, bilateral percutaneous access was used in 52.5% of cases (53/99). Access-related complications requiring surgical conversion occurred in only two cases. Excluding the LIFE trial, where all cases had planned percutaneous access, bilateral percutaneous access was achieved in 39% of patients within the EU PMR cohort [13]. Comparatively, successful bilateral percutaneous access was utilized in only 17% of cases in the European module of the Global Registry for Endovascular Aortic Treatment of EVAR with the Gore Excluder (W. L. Gore and Associates) [19]. However, this result appears consistent with the findings from the Society of Vascular Surgery Vascular Quality Initiative Database, which includes multiple devices, where 64% of EVARs performed between 2014 and 2017 were carried out with percutaneous access [20].

In terms of secondary benefits, the polymer-based sealing mechanism allows for the effective treatment of proximal necks with complex, irregular anatomy. In this retrospective study, 45.54% of patients had a complex aortic neck.

These results are significant because nearly 41% of the patients enrolled in the Ovation pivotal study had a challenging anatomy that would place them outside the indications for use (IFU) with other stent grafts [7]. In a series of 106 EVAR patients evaluated against the IFU of various stent graft manufacturers, 72% of patients were anatomically eligible for Ovation, compared with only 59% for Endurant, 55% for Excluder, 36% for Zenith, and 35% for Aorfix (Lombard, Oxfordshire, United Kingdom) [21]. Similarly, Patelis et al. [22] reported a 78.9% suitability rate for Ovation, compared with 57.9% for Excluder and 52.6% for Zenith.

Achieving an adequate and durable seal between the stent graft and the aortic wall remains a challenge, especially in patients with short and irregular aortic necks. As outlined in the Society for Vascular Surgery (SVS) AAA and in the European Society for Vascular Surgery (ESVS) Clinical Practice Guidelines, consistent long-term surveillance is essential to detect endoleaks after EVAR, especially type IA endoleaks [23,24].

EVAR devices are typically oversized by 15% to 30% to ensure proper apposition of the stent graft to the aortic wall. This results in continuous outward radial force being applied to the aneurysm neck. In a meta-analysis of neck dilation after EVAR, patients with neck dilation were more likely to develop type IA endoleak, experience graft migration, or require reintervention [25].

In this retrospective study, no late type IA endoleaks or stent graft migrations were documented during follow-up. The proximal seal of the Ovation device does not rely on outward radial force. The durability of the proximal seal of the Ovation device may be attributed to the use of polymer in the proximal seal zone, in contrast to self-expanding stents. The sealing rings in the proximal seal zone are filled with polymer and conform to the patient’s neck anatomy. Once the polymer sets, no outward force is exerted on the aneurysm neck. In an Italian series of patients undergoing EVAR with the Ovation device, no neck dilation was observed in most patients at 2 years post-procedure. Minimal chronic outward force seems to prevent further aortic neck dilation over time [17]. Regarding the Ovation device, de Donato et al. report freedom from type Ia endoleak at 98% for AAA aortic neck lengths > 7 mm and 96.8% for neck lengths < 7 mm, respectively [26].

However, further research is required to determine whether post-EVAR neck dilation and subsequent complications at the proximal neck can be mitigated by the Ovation platform’s polymer-based proximal seal. The superiority of a specific sealing concept can only be confirmed through a randomized trial.

The design of the Ovation device does introduce the risk of polymer leakage. In this retrospective analysis, one graft-related polymer leak event was documented: a patient experienced an anaphylactic reaction following a polymer leak outside the device circuit. The procedure was completed after hemodynamic stabilization. By the second postoperative day, the patient reported a loss of leg strength. Spinal cord injury was ruled out through electromyographic testing, and the clinical signs were attributed to muscle necrosis and peripheral nerve involvement following arterial embolization [27].

The literature contains few reports describing an anaphylactic reaction due to the leakage of the polymer outside the filling channels, with effects ranging from transient hypotension to more severe cardiovascular collapse [28,29]. As detailed in the publication of the 1-year IDE results, a root cause analysis of this complication was conducted, which led to a redesign of the polymer delivery system [13].

The results of this study must be interpreted within the context of its design. It is a retrospective analysis of prospectively obtained data. Treatment with the Ovation platform was assigned using a non-randomized approach. This analysis is purely descriptive, reporting mid- and long-term outcomes after EVAR with the Ovation platform in a single institution.

## 5. Conclusions

The results demonstrate durable aneurysm exclusion with the Ovation stent graft, even in complex anatomies, particularly in patients with small, tortuous vascular accesses and diseased proximal necks. However, in this series, the occurrence of a significant rate of graft-related adverse events indicated the necessity for structural improvements, which were implemented in the next-generation devices.

## Figures and Tables

**Table 1:** Patient’s baseline characteristics. Note: data are n (%) unless otherwise indicated.

Patients’ Baseline Characteristics	
Age, years	**73 ± 7.26** yrs
Male sex	**87/99 (86.10%)**
Hypertension	**84/99 (83.16%)**
COPD	**30/99 (29.70%)**
Diabetes mellitus	**27/99 (26.73%)**
Dyslipidaemia	**27/99 (26.73%)**
Previous coronary artery disease	**51/99 (50.49%)**
Smoking history	**84/99 (83.16%)**
Aneurysm diameter (mean ± std)	**53.7 ± 8.8 mm**

**Table 2:** Baseline aneurysm characteristics. Note: data are n (%) unless otherwise indicated.

Baseline Aneurysm Characteristics Study Cohort (n = 99)	
AAA diameter, mm	**53.7 ± 8.8**
Proximal neck diameter, mm • Suprarenal • Infrarenal	**24.04 ± 2.55** **23.12 ± 3.00**
Proximal neck length, mm	**23.93 ± 10.80**
Neck thrombosis	**32/99 (31.7%)**
Hostile neck	**46/99 (45.54%)**
Right CIA diameter, mm	**13.96 ± 5.14**
Left CIA diameter, mm	**13.71 ± 3.89**
Right EIA diameter, mm	**9.18 ± 1.88**
Left EIA diameter, mm	**9.25 ± 1.67**
Iliac calcification	**45/99 (44.55%)**

**Table 3:** Procedural data and perioperative results. Note: data are n (%) unless otherwise indicated. OSR = open surgical repair.

Procedural Data and Perioperative Results	
Procedure time, min	**82.13** ± **29.85** min
Fluoroscopy time, min	**22.11** ± **15.54** min
CMV, mL	**107.22** ± **27.83** mL
Percutaneous bilateral access	**53/99 (52.47%)**
Technical success	**94/99 (93.06%)**
Intraoperative death	**0/99 (0.00%)**
Intraoperative conversion to OSR	**0/99 (0.00%)**
Access surgical conversion	**2/99 (1.98%)**
Perioperative mortality	**4/99 (3.96%)**

**Table 4:** Patients requiring reintervention.

Indication for Reintervention	n° Patients (tot. 99)
**Type II endoleak**	**9 (8.90%)**
**Limb occlusion**	**2 (1.98%)**
**Type IA endoleak**	**0 (0.00%)**
**Type IB endoleak**	**5 (4.95%)**
**Para-visceral PAU/Para-visceral Pseudoaneurysm**	**2 (1.98%)**

## Data Availability

All data supporting the findings of this study are included within the article.
